# Cardiotoxicity in Adult Patients with Relapsed or Refractory Acute Myeloid Leukemia

**DOI:** 10.3390/cancers17152413

**Published:** 2025-07-22

**Authors:** Laura Torres-Miñana, Blanca Boluda, Antonio Solana-Altabella, Rebeca Rodríguez-Veiga, Isabel Cano, Evelyn Acuña-Cruz, Irene Navarro-Vicente, Pilar Lloret-Madrid, Paulina Hillebrand, David Martínez-Campuzano, Ana Osa-Sáez, Jaume Aguero, Yolanda Mendizábal, Beatriz Martín-Herreros, Eva Barragán, Claudia Sargas, Cristina Gil, Carmen Botella, Lorenzo Algarra, José Santiago Bermon, Raimundo García Boyero, María José Sayas, Mar Tormo, Aurelio López, Marta Valero-Nuñez, Marisa Calabuig, Javier De la Rubia, David Martínez-Cuadrón, Pau Montesinos

**Affiliations:** 1 Hematology Department, Hospital Universitari i Politècnic La Fe, 46026 València, Spain; laura_torres@iislafe.es (L.T.-M.); blanca_boluda@iislafe.es (B.B.); isabel_cano@iislafe.es (I.C.); evelyn_acuna@iislafe.es (E.A.-C.); irene_navarro@iislafe.es (I.N.-V.); pilar_lloret@iislafe.es (P.L.-M.); paulina_hillebrand@iislafe.es (P.H.); david_campuzano@iislafe.es (D.M.-C.); yolanda_mendizabal@iislafe.es (Y.M.); beatriz_martin@iislafe.es (B.M.-H.); barragan_eva@gva.es (E.B.); claudia_sargas@iislafe.es (C.S.); delarubia_jav@gva.es (J.D.l.R.); david_martinez@iislafe.es (D.M.-C.); montesinos_pau@gva.es (P.M.); 2 Instituto de Investigación Sanitaria La Fe, 46026 València, Spain; solana_ant@gva.es; 3 Pharmacy Department, Hospital Universitari i Politècnic La Fe, 46026 València, Spain; 4 Cardiology Department, Hospital Universitari i Politècnic La Fe, 46026 València, Spain; osa_ana@gva.es (A.O.-S.); aguero_jai@gva.es (J.A.); 5 CIBERONC CB16/12/00284, 46001 València, Spain; 6 Hematology Department, Hospital General de Alicante, 03010 Alicante, Spain; gil_cricor@gva.es (C.G.); botella_marpri@gva.es (C.B.); 7 Hematology Department, Hospital General Universitario, 02006 Albacete, Spain; jlalgarra@sescam.jccm.es (L.A.); jsantiago@sescam.jccm.es (J.S.B.); 8 Hematology Department, Hospital Universitario General de Castellón, 12004 Castellón de la Plana, Spain; garcia_rai@gva.es; 9 Hematology Department, Hospital Universitario Dr Peset, 46017 València, Spain; sayas_mjo@gva.es; 10 Hematology Department, Hospital Clínico Universitario–INCLIVA de Valencia, 46010 València, Spain; tormo_mar@gva.es (M.T.); calabuig_marmun@gva.es (M.C.); 11 Hematology Department, Hospital Arnau de Vilanova, 46015 València, Spain; lopez_aurmar@gva.es (A.L.); valero_marnun@gva.es (M.V.-N.); 12 Haematology Department, Universidad Católica “San Vicente Mártir”, 46001 València, Spain; 13 Department of Medicine, Universitat de València, 46010 València, Spain

**Keywords:** cardiotoxicity, relapse refractory AML, real-world, risk factors, outcomes

## Abstract

Background/Objectives: The incidence of cardiac morbimortality in patients with relapsed/refractory (R/R) acute myeloid leukemia (AML) is unknown. Methods: We analyze the characteristics, incidence, risk factors, and outcomes of cardiac events in AML patients treated for second-line (2L) or third-line (3L) episodes. Results: Among 327 2L AML patients (median age 62 years old), 135 experienced cardiac events, with an incidence of 38.6% non-fatal and 1.3% fatal events at 6 months. The grade 1–2 incidence was 16.8%, and the grade 3–4 incidence was 23.5% at 6 months. Overall, 207 cardiac events occurred in the 2L cohort, the most frequent being hypertension (n = 45), bradycardia (n = 39), QTc prolongation (n = 35), heart failure (n = 33), syncope/presyncope (n = 22), arrhythmia (n = 18), and myocardial ischemia (n = 8). Median OS in the 2L cohort was 9.4 months, 21.4 months in patients with grade 1–2, 8.8 months in patients without a cardiac event, 7.6 months in grade 3–4 patients, and 2.1 months with in 5 patients (*p* = 0.0035). The multivariate analysis showed prior cardiologic antecedents (*p* = 0.013), intensive 2L chemotherapy (*p* = 0.01), and inclusion in a 2L clinical trial (*p* < 0.001) as independent risk factors for non-fatal cardiac events. Among 189 patients of the 3L cohort, the incidence of non-fatal and fatal cardiac events was 49.2% and 0% at 6 months, respectively. Non-fatal cardiac events were more frequent in patients with prior cardiac antecedents (*p* = 0.004). Conclusions: In summary, cardiotoxicity is a frequent and challenging complication in R/R AML patients. We identified the risk factors that could be relevant to implementing risk-adapted management guidelines, aiming to reduce morbi-mortality in this difficult-to-treat setting.

## Simple Summary

The incidence of cardiac morbidity and mortality in patients with relapsed/refractory (R/R) acute myeloid leukemia (AML) is unknown. We aim to estimate the cumulative incidence (CI) of cardiac events in R/R AML patients and to identify the risk factors for their occurrence. In this study, we found a 38.6% incidence of non-fatal cardiac events in second-line (2L) and 49.2% in third-line (3L) treatments, and 1.3% of fatal cardiac events in 2L and 0% in 3L treatments. The analysis identified the following independent risk factors for non-fatal cardiac events: prior cardiac history (*p* = 0.013), intensive 2L chemotherapy (*p* = 0.01), and inclusion in a 2L clinical trial (*p* < 0.001). In summary, cardiotoxicity is a frequent and challenging complication in R/R AML patients.

## 1. Introduction

Despite the therapeutic improvements made in recent years, many patients with acute myeloid leukemia (AML) will experience at least one relapsed/refractory (R/R) episode, and their prognosis in this situation is dismal [1,2,3,4]. R/R AML patients receive potentially cardiotoxic drugs [5,6,7,8,9], such as anthracyclines [4,10,11], which may cause anthracycline-related left ventricular dysfunction [8,12], and drugs leading to QT interval prolongation (QTc) [13] (e.g., FLT3 inhibitors [14,15,16], or IDH inhibitors [17,18], among others) [11,19,20,21,22]. However, there is still no “standard” definition of cardiotoxicity [5,12,20,23], and most studies on AML have focused on the first-line setting [24] and on the pediatric population [19,25,26], making it difficult to estimate the rate and severity of cardiac complications among adult R/R patients. Some studies have analyzed the impact of upfront anthracycline-based regimens [27], enabling individualized approaches in this setting [2,12,28,29], but scarce data are available on other cardiac events. As far as we know, no previous study has provided a comprehensive overview of cardiac toxicities in a real-world series of patients with R/R AML [5,6,12]. Such analyses could be useful to estimate the incidence, outcomes, and risk factors for cardiac toxicities in this very difficult-to-treat population, often receiving experimental approaches in the context of clinical trials or as compassionate use.

The aim of this study is to estimate the incidence of cardiac events in a large series of patients with R/R AML. We also aim to identify the risk factors associated with increased rates of fatal and non-fatal cardiac events. This study sheds light on the current real-world incidence and outcomes of cardiac events in patients with R/R AML.

## 2. Materials and Methods

### 2.1. Study Design and Population

We performed a systematic and retrospective medical record review of all patients diagnosed with AML who were treated receiving second-line (2L) or third-line (3L) therapy between 1 January 2011 and 31 June 2020 at the HULaFe facility in Valencia, Spain. Patients diagnosed with acute promyelocytic leukemia and those aged less than 18 years old at R/R diagnosis (i.e., index date) were excluded. In accordance with the principles outlined in the Declaration of Helsinki, the protocol was approved by the local Clinical Research Ethics Committee.

### 2.2. Study Objectives and Variables

The 2L cohort was composed of adult AML patients with first R/R treated at HULaFe. The observation period began from the first R/R episode date to the second R/R episode date, last follow-up, or death, whichever occurred first. The 3L cohort was composed of AML adult patients in the second R/R episode. For this cohort, the observation period ran from the second R/R episode to the date of last follow-up or death, whichever occurred first.

Our primary objective was to describe the overall incidence of fatal and non-fatal cardiac events in AML patients in the 2L and 3L cohorts. The secondary objectives were as follows: (1) analyze risk factors for the development of fatal and non-fatal cardiac events in the 2L and 3L cohort, (2) describe and categorize cardiac events, and (3) describe response rates (complete remission [CR], complete remission with incomplete hematologic recovery [CRi]) and long-term outcomes (overall survival [OS], event-free survival [EFS]) according to the occurrence of cardiac events.

The following information was collected at index date: age; gender; cardiac and other co-morbidities; bone marrow assessment (blasts percentage); cytogenetic risk stratification; gene mutations (FLT3-ITD, FLT3-TKD, IDH, NPM1); Eastern Cooperative Oncology Group (ECOG) scale; de novo or secondary AML; baseline cardiologic medication; cumulative dose of anthracyclines; and the treatment regimen, including investigational therapies, in the 2L or 3L groups.

Variables related to cardiac events were collected: type and grade of cardiac event according to Common Terminology Criteria for Adverse Events Version 5.0 (CTCAE V5) [30,31], date of cardiac event, admission for the event (inpatient/outpatient, domiciliary hospitalization, intensive care admission), outcome of the cardiac event, and concomitant medications or other causes contributing to cardiac complications.

All echocardiograms, blood tests, electrocardiograms (ECGs), and vital signs, focusing on heart rate and blood pressure, were reviewed from the index date. Death causality was assigned to a cardiac event or to another cause according to the investigator’s clinical judgment.

### 2.3. Definitions

Treatments were classified as intensive (i.e., cytarabine and idarubicin like 3 + 7) or low-intensity therapy (i.e., hypomethylating agents in monotherapy or in combination, low-dose cytarabine-based regimens, or FLT3 inhibitors as monotherapy).

Refractory AML was defined as a no CR/CRi after two cycles of intensive chemotherapy [32], excluding deaths in aplasia. Patients treated with low-intensity therapy were classified as refractory disease when they showed leukemic progression, lack of clinical benefit, or switched to another therapy. Relapse was defined as AML subjects who achieved a CR/CRi with a prior line of treatment and showed reappearance of >5% of blasts in the bone marrow and/or peripheral blood (excluding regenerative blasts), or extramedullary disease.

The following cardiac events were registered: (1) Myocardial ischemic events (according to CTCAE) [30]. (2) Arrhythmia (i.e., atrial fibrillation, atrial flutter, complete atrioventricular (AV) block, and others). (3) Heart failure and its related complications. (4) QT prolongation in more than one assessment. (5) Sinus bradycardia (if heart rate <55 beats per minute and/or considered to be clinically significant). (6) Sinus tachycardia (if considered to be clinically significant). Sinus tachycardia in the context of sepsis or fever or moderate/severe anemia was not considered as a cardiac event. (7) Hypotension (if considered clinically relevant and not explained by sepsis or fever or anemia). (8) Hypertension (if grade 3 requiring addition of antihypertensive treatment or if considered clinically relevant). (9) Other (e.g., syncope, presyncope, pericarditis, valve disease, tamponade, other ECG abnormalities).

Regarding cardiac antecedents, we defined a category of clinically relevant cardiac antecedents. For more details, these definitions are also reported in the previous manuscript by Boluda et al. [24].

Pre-treatment echocardiogram for the ejection fraction was not performed routinely at baseline, but it was indicated in patients when clinically indicated or required by protocol. The QTc interval was calculated using the Fridericia formula (QTcF).

Cardiac history was considered in its own category due to the worse prognosis and severity of these patients.

### 2.4. Statistical Analyses

The statistical analysis was performed following the same approach as described in the previous manuscript of Boluda et al. (Boluda, B. et al., ‘Incidence and Risk Factors for Development of Cardiac Toxicity in Adult Patients with Newly Diagnosed Acute Myeloid Leukemia’. *Cancers* (Basel). 2023, 15 (8).) [24]. Incidences and proportions were reported for continuous variables, and means or medians for categorical ones. To analyze differences in the distribution of variables between subsets of patients, in the non-parametric distribution variables, we used Chi-square with Yates’ correction, and Mann–Whitney U and Student’s *t*-tests for parametric variables. Patients presenting with several cardiac events were considered to have only one event for the purpose of calculating overall incidence. For non-fatal and overall cardiac events, the incidence was calculated accounting for the first cardiac event for each patient. In patients with several cardiac events, the most severe cardiac event was the first. The cumulative incidence of a cardiac event was calculated using the cumulative incidence method, and the first cardiac event was counted as an event (according to the defined category, i.e., fatal, non-fatal). For OS, an event was defined as death by any cause, while for EFS, it was failure to achieve CR/CRi, relapse after CR/CRi, or death by any cause, whichever occurred first. Time-to-event analyses were calculated from the first treatment after the index date (in months), and OS and EFS were summarized using Kaplan–Meier (KM) curves. For the univariate risk factor analyses (using the Gray test), we considered a significant association to have *p*-values < 0.05. The impact of risk factors for cardiac events was assessed using multivariate Cox proportional hazard regression. Analyses were conducted using R.2.14 statistical software.

## 3. Results

### 3.1. Patient and Disease Characteristics of the 2L and 3L Cohorts

**Figure 1:**
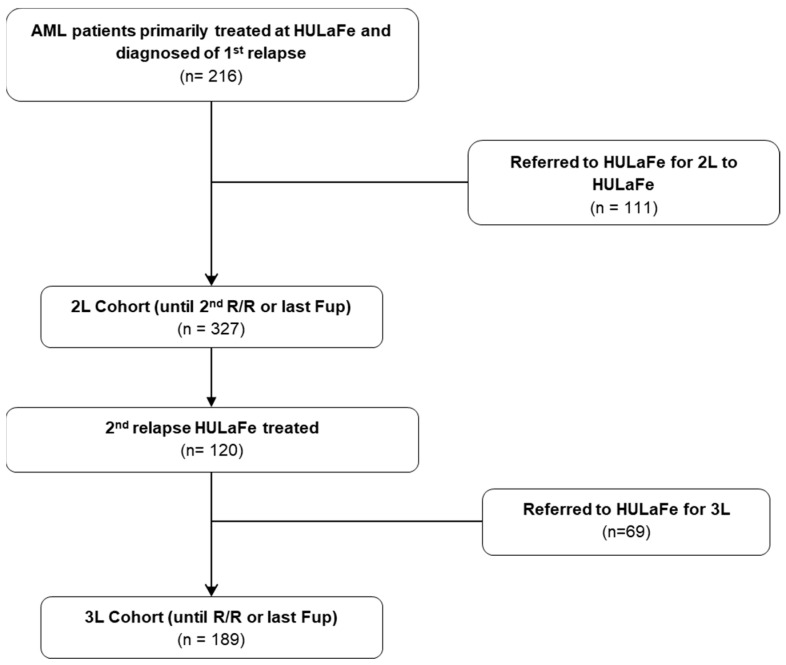
Consort diagram.

Overall, 327 patients with a first R/R AML episode (2L cohort) and 189 patients with a second R/R AML episode (3L cohort) were identified (of those, 120 were also patients included in the 2L cohort) (Figure 1). Median age of 2L patients was 62 years (range 21–87 years); 190 (58%) were male, 115 (35%) were receiving secondary or therapy-related AML, and 38 (12%) had FLT3-ITD mutation at the R/R time (Table 1). Overall, 72 patients (22%) received 2L intensive chemotherapy, 46 (14%) non-intensive therapy, and 209 (64%) were enrolled in a clinical trial (Table 2). Antecedent or ongoing relevant cardiac comorbidities were present in 35 patients (11%), and 112 (34%) had any kind of cardiac antecedent at the time of the first R/R AML (Table 3).

Median age of 3L patients was 58 years (range 20–87 years); 110 (58%) were male, 45 (24%) had secondary or therapy-related AML, and 20 (11%) had FLT3-ITD mutation (Table 1). Overall, 50 (26%) received intensive chemotherapy, 33 (17%) received non-intensive treatment, and 105 (57%) were included in a clinical trial (Table 2). Antecedent or ongoing relevant cardiac comorbidities were present in 10 patients (5%), and 67 (36%) had any kind of cardiac antecedent at the time of second R/R AML (Table 3). The median follow-up of patients alive in the 2L cohort was 1136 days, and 1933 days for the 3L cohort.

### 3.2. Incidence and Characteristics of Cardiac Events in the 2L Cohort

**Figure 2:**
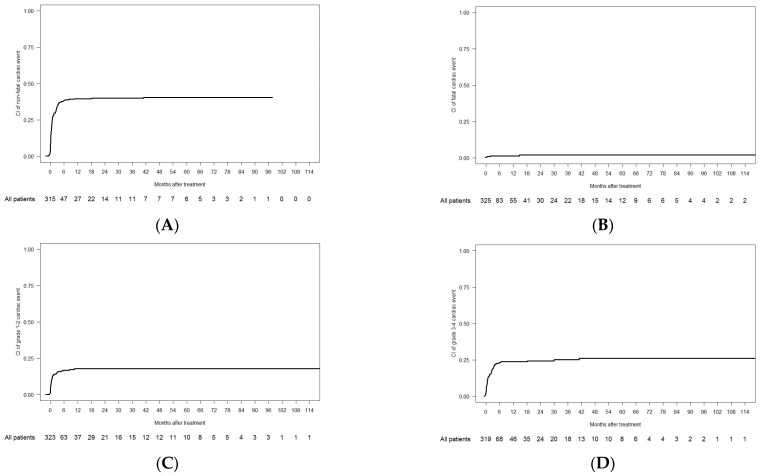
Cumulative incidence of cardiac events in the 2L cohort: (**A**) non-fatal; (**B**) fatal; (**C**) grade 1–2; and (**D**) grade 3–4.

Overall, 135 patients experienced cardiac events in the 2L observation period (crude incidence of 41.3%), and 5 of them were fatal (crude incidence of 1.5%). The CI of non-fatal cardiac events was 38.6% at 6 months and 40.5% at 5 years, and the CI of fatal events (i.e., grade 5) was 1.3% at 6 months and 2% at 5 years (Table 4 and Figure 2A,B). The CI of grade 1–2 cardiac events (n = 54) was 16.8% at 6 months and 17.8% at 5 years, and the CI of grade 3–4 events (n = 76) was 23.5% at 6 months and 26% at 5 years (Figure 2B–D).

Overall, 207 cardiac events occurred among 135 patients with at least one cardiac event, the most frequent being hypertension (n = 45), sinus bradycardia (n = 39), QTc prolongation (n = 35), heart failure (n = 33), syncope/presyncope (n = 22), arrhythmias (n = 18), and myocardial ischemia (n = 8). Among fatal cardiac events, three were heart failure, and two were arrhythmia (one ventricular fibrillation, one asystolia) (Supplementary Table S1).

### 3.3. Risk Factors for Development of Cardiac Events in the 2L Cohort

Univariate analysis showed that patients with prior cardiac antecedent (relevant and not relevant) (*p* = 0.036), with ECOG ≥ 2 (*p* = 0.036), receiving intensive therapies (*p* = 0.02), and those included in clinical trials (*p* < 0.001) had increased CI of non-fatal cardiac events. There was a trend for increased risk of fatal cardiac events in those <65 years old (*p* = 0.058) and with ECOG ≥ 2 (*p* = 0.084). An increased CI of grade 1–2 events was observed among patients previously treated with anthracycline (*p* = 0.046), with ECOG < 2 (*p* = 0.017), receiving intensive chemotherapy (*p* = 0.044), and included in 2L clinical trial (*p* < 0.001). No risk factors were significantly associated with grade 3–4 cardiac events (Table 4).

The multivariate analysis showed that any prior cardiological history (*p* = 0.013), intensive 2L chemotherapy (*p* = 0.01), and inclusion in a 2L clinical trial (*p* < 0.001) were independent risk factors for the development of non-fatal cardiac events (Supplementary Table S2).

### 3.4. Impact of Cardiac Events on Outcomes After Intensive Salvage Regimens (2L Cohort)

**Figure 3:**
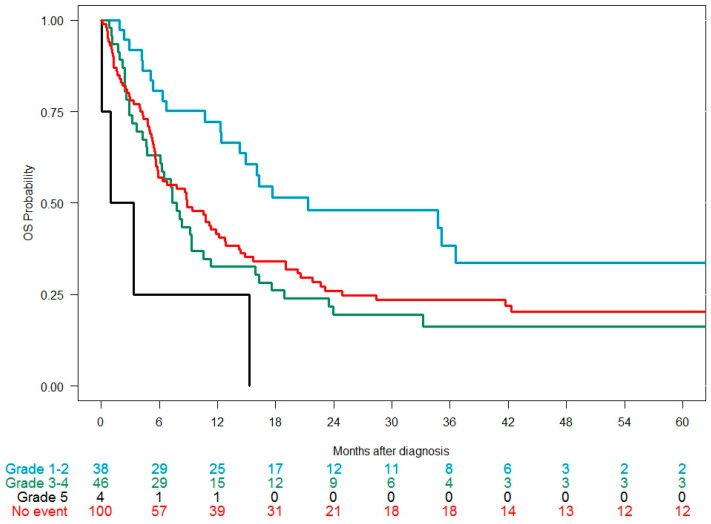
Overall survival in the 2L cohort (intensive salvage regimens only, n = 187) since the time of diagnosis of the relapse/refractory episode according to the CTCAE grade of the cardiac event developed during the observation period.

Among 187 patients receiving an intensive 2L regimen, the CR/CRi rate was 45.9%; 57.9% in patients developing a grade 1–2 cardiac event, 53.3% in those developing a grade 3–4 event, 39% in those without a cardiac event, and 25% in those with a grade 5 event (*p* = 0.021). Median OS was 9.4 months: 21.4 months with grade 1–2 events, 8.8 months in patients without a cardiac event, 7.6 months with grade 3–4 events, and 2.1 months with grade 5 events (*p* = 0.0035) (Figure 3). Median EFS was 1.9 months, with no differences according to cardiac events (Table 5).

### 3.5. Incidence and Characteristics of Cardiac Events in the 3L Cohort

Out of 189, 103 patients experienced cardiac events in the 3L cohort (crude incidence of 54.5), and 1 of them was fatal (crude incidence 0.5%). The CI of non-fatal cardiac events was 49.2% at 6 months and 54.2% at 5 years, and the CI of fatal events (i.e., grade 5) was 0% at 6 months and 1.4% at 5 years (Supplementary Table S2). The CI of grade 1–2 cardiac events (n = 37) was 17.6% at 6 months and 23.1% at 5 years, and the CI of grade 3–4 events (n = 65) was 32% at 6 months and 38.3% at 5 years.

Overall, 159 cardiac events occurred during the 3L observation period, the most frequent being QTc prolongation (n = 39), hypertension (n = 30), sinus bradycardia (n = 20), arrhythmia (n = 21), heart failure (n = 20), syncope/presyncope (n = 14), pericarditis/pericardial effusion (n = 8), and myocardial ischemia (n = 4) (Supplementary Table S3).

### 3.6. Risk Factors for Cardiac Events in the 3L Cohort

Non-fatal cardiac events were more frequent in 3L patients with prior cardiological antecedents (all type) (66.7% vs. 47.2%, *p* = 0.004) and grade 1–2 events among patients with prior anthracyclines (38.3% vs. 20.4%, *p* = 0.01) and those included in a 3L clinical trial (27.8% vs. 17.3%, *p* = 0.047). Prior cardiac antecedent (all types) was associated with increased CI of grade 3–4 cardiac events (48.4% vs. 33%, *p* = 0.04).

### 3.7. Impact of Cardiac Events on Outcomes After Intensive Salvage Regimens (3L Cohort)

Among 102 patients receiving an intensive 3L regimen, the CR/CRi rate was 32.6%: 33.3% in patients developing grade 1–2 cardiac events, 36.4% in those developing grade 3–4 events, 29.4% in those without cardiac events, and 0% in 1 patient with a grade 5 event (*p* = 0.021) (Table 5). Median OS was 5.4 months: 5.4 months in patients with grade 1–2 events, 5.9 months without a cardiac event, 5.0 months with grade 3–4 events, and not calculated with grade 5 events (*p* = 0.87); see Table 6.

## 4. Discussion

This study shows that the development of cardiac complications is very common in adult AML patients receiving salvage therapy for their first or second R/R episode. Reduced complete remission rates, EFS, and OS were observed among patients in first R/R salvage with grade 3–4 cardiotoxicity, while outcomes were not affected by development of grade 1–2 events and/or in second R/R salvage. Several risk factors may increase the risk of non-fatal cardiac events during the first R/R episode, such as prior cardiologic antecedents, intensive 2L chemotherapy (*p* = 0.01), and inclusion in a 2L clinical trial.

As far as we know, this is the first study assessing cardiotoxicity in a large real-world series of R/R AML patients. Previous studies on cardiotoxicity have focused on decreases in LVEF secondary to the use of anthracyclines in the first line of treatment [4,8,24,33], especially in the pediatric population [8,34,35,36,37,38]. Here, we analyze all potential cardiotoxicities in an R/R setting, showing that LVEF and heart failure represent less than 1/3 of cardiac complications. We reveal that arrhythmia, QTc prolongation, and other conduction disorders are especially frequent, and this could be explained by several issues: (1) The presence of metabolic disturbances triggering arrhythmogenic events. We show that roughly 40% of patients had such abnormalities at the time of the cardiac event. (2) Frequent use of drugs prolonging QTc interval, such as triazoles, which were used in more than half of patients at the time of the cardiac event. Also, FLT3 inhibitors or other small molecules (e.g., in clinical trials) were frequently used in our patient cohort. (3) Prior exposure to anthracyclines, which was frequent among the 2L (69%) and 3L cohort (83%)—in the later cohort, showing a median cumulative doxorubicin equivalent dosage of 330 mg/m^2^. (4) Other baseline conditions leading to an increased risk of cardiac events, present in 34% of 2L and 30% of 3L cohorts, such as prior LVEF reduction or heart failure and QTc abnormalities [39]. It should be noted that, among our 2L and 3L cohorts, prior cardiac antecedents were less frequent than in the study by Boluda et al. [24], carried out in first-line patients of our institution, possibly reflecting that younger and less comorbid patients are usually selected for 2L and 3L therapies. In addition, 64% and 57% of 2L and 3L patients, respectively, were treated in the context of clinical trials, which often require strict inclusion/exclusion selection criteria.

In our 2L cohort, we found a similar 6-month cumulative incidence of non-fatal and fatal cardiac events (38.6% and 1.3%, respectively) when compared to our previous report in first-line patients (37.8% and 1.2%, respectively) [24]. Also, we found that the vast majority of cardiotoxic complications occurred in the first months after starting salvage therapy, with residual cumulative cardiac events beyond 6 months. As in our previous first-line analysis, most cardiac events were graded as 3–4 according to the CTCAE classification, although the proportion of grade 1–2 events was also relevant. Regarding the 3L cohort, we found a slightly higher incidence of cardiac complications, probably due to the expanded observation period in this cohort (until death or last follow-up, without ceasing at the subsequent R/R episode).

Among patients receiving intensive 2L chemotherapy, the development of cardiac events was not associated with lower CR/CRi rates (except for those at grade 5), probably because patients achieving a response had a greater time of exposure and risk of developing complications. However, we found that grade 3–4 cardiotoxic events led to decreased EFS and OS, while grade 1–2 did not impact them. In this regard, we should note that most grade 1–2 toxicities corresponded to QTc prolongation, sinus bradycardia, and presyncope, while grade 3–4 events were truly life-threatening (e.g., heart failure, atrial fibrillation, uncontrolled hypertension, and arrhythmia).

We identified several risk factors for the development of non-fatal cardiac toxicity among R/R AML patients, which could be useful to implement risk-adapted management strategies. As previously described for first-line AML, prior cardiologic antecedents and inclusion in clinical trials were associated with an increased risk factor [24,40]. We can speculate that patients enrolled in clinical trials could be tightly monitored and followed up, increasing the performance of complementary procedures, such as ECGs, and, therefore, the detection of complications. Treatment with intensive 2L chemotherapy was also an independent risk factor, which could be related to anthracycline administration and higher CR/CRi rates.

Our study is limited by its retrospective and single-institution design, and we did not perform a systematic baseline echocardiogram or ECG in all patients, reflecting real-life practice. However, data were collected and interpreted by experienced physicians, in contrast to industry-sponsored trials where other staff are frequently responsible for these tasks. We acknowledge that comparing the OS and EFS outcomes between patients developing or not developing cardiac events could be difficult to interpret, as there is a time bias for patients developing cardiac events whereby they could have inherently more time exposure to develop these complications. However, we can infer from our data that developing a grade 1–2 cardiac event does not harm prognosis, while developing grade 3–4 does.

## 5. Conclusions

In conclusion, cardiotoxicity is a frequent and challenging complication in salvage treatment of R/R AML. We identify several risk factors and underlying conditions which could be relevant to implementing risk-adapted management guidelines, aiming to reduce morbidity and mortality in this difficult-to-treat population.

## Figures and Tables

**Table 1:** Baseline characteristics of adult AML patients according to line of treatment (2L and 3L).

Characteristic	2L Cohort		3L Cohort	
	Median (Range)	n (%)	Median (Range)	n (%)
N		327		189
Age, years	62 (21–87)	327	58 (20–87)	189
<65		187 (57)		125 (66)
≥65		140 (43)		64 (34)
Gender		327		189
Male		190 (58)		110 (58)
Female		137 (42)		79 (42)
ECOG status		320		180
0–1		273 (85)		158 (87)
≥2		47 (15)		22 (13)
Type of acute myeloid leukemia		327		189
De novo		211 (65)		144 (76)
Therapy related		43 (13)		17 (9)
Previous MDS/MPN		72 (22)		28 (15)
Extramedullary disease		327		189
Yes		19 (6)		19 (10)
No		308 (94)		170 (90)
White blood cell count in peripheral blood, ×10^9^/L	2.7 (0.1–156.9)	323	2.9 (0.2–253)	188
Hemoglobin, g/dL	9.8 (5–16.2)	322	9.4 (5.9–15.5)	188
Platelet count, ×10^9^/L	52 (1–704)	322	31 (2–751)	188
Creatinine, mg/dL	0.8 (0.2–3.3)	321	0.8 (0.2–3.4)	187
Bilirubin, mg/dL	0.5 (0.1–5.5)	312	0.5 (0.2–2.4)	180
Albumin, g/dL	4 (2.1–5.5)	282	4 (2.3–5)	174
Lactate dehydrogenase, U/L	339 (82–16,666)	300	314 (83–5665)	183
MRC Cytogenetic risk		121		45
Favorable		0 (80)		1 (2)
Intermediate		58 (48)		30 (65)
Adverse		63 (52)		14 (33)
FLT3-ITD mutation		175		76
Positive		38 (22)		20 (26)
Negative		137 (78)		56 (74)
FLT3-TKD mutation		165		74
Positive		17 (10)		9 (12)
Negative		148 (90)		65 (88)
NPM1 mutation		149		77
Positive		49 (33)		24 (31)
Negative		100 (66)		53 (69)
IDH mutation		234		64
IDH1 positive		15 (6)		9 (14)
IDH2 positive		23 (10)		18 (28)
Negative		196 (84)		37 (58)

Abbreviations: FLT3: Fms-like tyrosine kinase 3; ITD: internal tandem duplication; TKD: tyrosine kinase domain; ECOG: Eastern oncology cooperative group; MDS: myelodysplastic syndrome; MPN: chronic myeloproiferative neoplasm; MRC: myeloid research council; NPM1: nucleophosmin 1; IDH: isocitrate dehydrogenase.

**Table 2:** Therapeutic approach among 1R/2R AML patients (intensive vs. non-intensive-based vs. clinical trial).

Therapeutic Approach	Schedule	2L Cohort Number of Patients n = 327 (%)	3L Cohort Number of Patients n = 189 (%)
**Intensive chemotherapy**		**72 (22)**	**50 (26)**
	IDA + Ara-C (3 + 7)	10 (3)	2 (1)
	Ara-C	2 (1)	0
	FLAG-IDA (fludarabine + Ara-C + IDA)	53 (16)	31 (16)
	EMA (mitoxantrone + Ara-C + etoposide)	1 (0.3)	2 (1)
	Allogeneic transplant	6 (2)	15 (8)
**Non-intensive therapy**		**46 (14)**	**33 (17)**
	Azacitidine	6 (2)	3 (2)
	Decitabine	2 (1)	2 (1)
	LD-Ara-C or FLUGA (fludarabine + LD-Ara-C)	34 (10)	20 (10)
	Gilteritinib/quizartinib/other FLT3 inhibitor in monotherapy	1 (0.3)	3 (2)
	Other non-intensive	3 (1)	5 (3)
**Clinical Trial**		**209 (64)**	**107 (57)**
	Intensive + FLT3 inhibitor/placebo	15 (5)	1 (1)
	Intensive without FLT3 inhibitor	100 (31)	50 (26)
	Gilteritinib/quizartinib/other FLT3 inhibitor in monotherapy	12 (4)	6 (3)
	Non-intensive without FLT3 inhibitor	82 (25)	49 (26)

Abbreviations: FLT3: Fms-like tyrosine kinase 3; LD: low dose; IDA: idarubicin.

**Table 3:** Baseline cardiac characteristics in all patients according to line of treatment (2L vs. 3L).

Characteristic	2L Cohort		3L Cohort	
	Median (Range)	Number of Patients n = 327 (%)	Median (Range)	Number of Patients n = 189 (%)
Relevant cardiac comorbidity		327		189
Yes		35 (11)		10 (5)
No		292 (89)		179 (95)
All cardiac comorbidity		327		189
Yes		112 (34)		67 (36)
No		215 (66)		122 (64)
Prior anthracycline		327		189
Yes		227 (69)		158 (83)
No		100 (31)		31 (17)
Prior dose of anthracycline, mg/m^2^	180 (60–660)		330 (120–990)	
Baseline cardiac medication		327		189
Yes		110 (34)		56 (30)
No		217 (66)		133 (70)
Baseline cardiac medication	0 (0–6)	327		189
0		217 (66)		133 (70)
1–2		78 (24)		42 (23)
3–4		25 (8)		13 (7)
>4		5 (2)		1 (1)
Baseline electrocardiogram		327		189
Normal		153 (47)		93 (49)
Abnormal non clinically significant		62 (19)		42 (23)
Abnormal clinically significant		7 (2)		3 (2)
Not available		105 (32)		51 (27)
Baseline QTcF electrocardiogram	418 (308–521)	217	420 (372–489)	137
QTcF < 450		201 (93)		125 (91)
QTcF 450–480		13 (6)		10 (7)
QTcF 481–500		2 (1)		2 (1)
QTcF > 500		1 (0.4)		0
Baseline echocardiogram		327		189
Normal		82 (25)		56 (29)
Abnormal		16 (5)		10 (5)
Not available		229 (70)		123 (65)
Baseline ejection fraction	65 (43–87)	87		56
LVEF < 50		2 (2)		0
LVEF ≥ 50		85 (98)		56 (100)
Heart failure at diagnosis		327		189
Yes		8 (2)		2 (1)
No		319 (98)		187 (99)
Baseline NYHA	0 (0–3)	323	0 (0–1)	189
0		319 (98)		187 (99)
1		5 (2)		2 (1)
2		2 (1)		0
3		1 (0.3)		0
4		0		0
Cardioischemic signs or symptoms at diagnosis		327		188
Yes		3 (1)		1 (1)
No		324 (99)		187 (99)

Abbreviations: LVEF: left ventricle ejection fraction; NYHA: New York Heart disease Association.

**Table 4:** Cardiac events in the 2L cohort (from first refractory/relapse to second refractory/relapse): crude and cumulative incidence of cardiac events (fatal vs. non-fatal vs. no cardiac event) according to the demographic, clinical, and biological characteristics of patients.

	No Cardiac Event	*p* Value	Fatal Cardiac Event				Non-Fatal Cardiac Event			
Characteristic	N (%)		N (%)	Cumulative Incidence			N (%)	Cumulative Incidence		
				At 6 Months, %	At Last FU, %	*p*		At 6 Months, %	At Last FU, %	*p*
**N**	192 (58.7)		5 (1.5)	1.3	2		130 (39.8)	38.6	40.5	
**Age**	192		5				130			
** <65 years**	104 (55.6)	0.087	5 (2.7)	2.3	3.4	0.058	78 (41.7)	40.2	42.6	0.36
** ≥65 years**	88 (62.9)		0	0	0		52 (37.1)	36.4	37.1	
**Relevant cardiologic antecedents**	192		5				130			
** No**	174 (59.6)	0.56	4 (1.4)	1.1	1.9	0.45	114 (39)	37.6	39.8	0.2
** Yes**	18 (51.4)		1 (2.9)	2.9	2.9		16 (45.7)	46.8	46.8	
**All cardiologic antecedents**	192		5				130			
** No**	135 (62.8)	0.12	3 (1.4)	1	1.9	0.7	77 (35.8)	35.1	36.2	0.036
** Yes**	57 (50.9)		2 (1.8)	1.8	1.8		53 (47.3)	45.2	47.1	
**Previous anthracycline treatment**	192		5				130			
** No**	66 (66)	0.09	0	0	0	0.15	34 (34)	33	33	0.14
** Yes**	126 (55.5)		5 (2.2)	1.9	2.8		96 (42.3)	41.1	43	
**ECOG at diagnosis**	186		5				129			
** <2**	153 (56)	0.03	3 (1.1)	0.7	1.6	0.084	117 (42.9)	41.6	43.6	0.036
** ≥2**	33 (70.2)		2 (4.3)	4.6	4.6		12 (25.5)	24.2	26.5	
**FLT3-ITD status**	90		4				81			
** Negative**	72 (52.6)	0.22	2 (1.5)	0.9	2.8	0.35	63 (46)	44.6	46.1	0.22
** Positive**	18 (47.4)		2 (5.3)	5.3	5.3		18 (47.4)	49.7	49.7	
**Treatment chemotherapy**	192		5				130			
** Intensive**	100 (53.2)	0.049	4 (2.1)	1.7	2.9	0.3	84 (44.7)	43.5	45.4	0.02
** Non-intensive**	92 (66.2)		1 (0.7)	0.7	0.7		46 (33.1)	31.9	33.3	
**Inclusion in clinical trial**	192		5				130			
** No**	84 (71.2)	<0.001	3 (2.5)	1.7	3.2	0.33	31 (26.3)	24.1	27.2	<0.001
** Yes**	108 (51.7)		2 (1)	1.1	1.1		99 (47.4)	46.7	47.6	
**Use of FLT3 inhibitors**	192		5				130			
** No**	173 (57.9)	0.51	5 (1.7)	1.4	2.2	0.46	121 (40.5)	39.2	41.1	0.26
** Yes**	19 (67.9)		0	0	0		9 (32.1)	32.1	32.1	

Abbreviations: FLT3: Fms-like tyrosine kinase 3; ECOG: Eastern oncology cooperative group.

**Table 5:** Main clinical outcomes (CR/CRi/OS/EFS) in 2L study cohort patients treated with intensive chemotherapy salvage according to the occurrence of cardiac events (grade 0–1–2 vs. 3–4 vs. 5; from first refractory/relapse episode to second refractory/relapse episode).

	All Patients	No Cardiac Event	Cardiac Event			*p*
			Grade 1–2	Grade 3–4	Grade 5	
	N (%)	N (%)	N (%)	N (%)	N (%)	
**Response, n (%)**	187 (100)	100 (100)	38 (100)	45 (100)	4 (100)	
** ORR (CR + CRi)**	86 (45.9)	39 (39)	22 (57.9)	24 (53.3)	1 (25)	0.021
** PR**	8 (4.3)	5 (5)	2 (5.3)	1 (2.2)	0 (0)	
** Resistance**	73 (39)	43 (43)	14 (36.8)	15 (33.3)	1 (25)	
** Induction death**	20 (10.7)	13 (13)	0 (0)	5 (11.1)	2 (50)	
**EFS, n (%)**	112	66 (58.9)	23 (20.5)	23 (20.5)	NA	
** Median (CI95), months**	1.9 (1.7–2.7)	1.9 (1.4–2.7)	1.8 (1.7–6.6)	2.4 (1.5–5.2)	NA	0.97
** 1 year (CI95), %**	9 (5–16)	9 (4–20)	13 (4.5–37.5)	8.7 (2.3–32.7)	NA	
** 3 year (CI95), %**	1 (0.1–6)	2.1 (0.8–11.9)	NA	NA	NA	
** 5 years (CI95), %**	0 (NA)	1.5 (0.2–10.6)	NA	NA	NA	
**OS, n (%)**	189	100	38	46	5	
** Median (CI95), months**	9.4 (7.8–12.9)	8.8 (5.9–12.8)	21.4 (14.3–NA)	7.6 (6.1–11.3)	2.1 (0.1–NA)	0.003
** 1 year (CI95), %**	44 (38–52)	42 (33–53)	72 (59–89)	33 (22–49)	25 (5–NA)	
** 3 year (CI95), %**	23 (18–31)	23 (16–34)	38 (24–61)	16 (8–32)	NA	
** 5 years (CI95), %**	20 (14–29)	20 (13–31)	34 (20–58)	16 (8–32)	NA	

ORR: overall response rate; CR: complete remission; CRi: complete remission with incomplete recovery; OS: overall survival; EFS: event-free survival; CI: confidence interval.

**Table 6:** Main clinical outcomes (CR/CRi/OS/EFS) in 3L study cohort patients treated with intensive chemotherapy salvage according to the occurrence of cardiac events (grade 0–1–2 vs. 3–4 vs. 5; from second refractory/relapse episode).

	All Patients	No Cardiac Event	Cardiac Event			*p*
			Grade 1–2	Grade 3–4	Grade 5	
	N (%)	N (%)	N (%)	N (%)	N (%)	
**Response, n (%)**	102 (100)	51 (100)	18 (100)	33 (100)	NA	
** ORR (CR + CRi)**	33 (32.6)	15 (29.4)	6 (33.3)	12 (36.4)	NA	NA
** PR**	4 (3.9)	2 (3.9)	1 (5.6)	1 (3)	NA	
** Resistance**	51 (50.5)	25 (49)	11 (61.1)	15 (45.5)	NA	
** Induction death**	14 (13.7)	9 (17.6)	0 (0)	5 (15.2)	NA	
**OS, n (%)**	102	51	18	33	NA	
** Median (CI95), months**	5.4 (4.6–8.1)	5.9 (3.9–10.4)	5.4 (3–21.5)	5 (4.2–19.3)	NA	0.84
** 1 year (CI95), %**	31.4 (23.5–41.8)	33.3 (22.6–49.1)	27.8 (13.2–58.5)	33.3 (20.6–54)	NA	
** 2 year (CI95), %**	20.6 (14.1–30.1)	23.5 (14.4–38.6)	16.7 (5.9–33.7)	21.2 (11–40.9)	NA	
** 3 year (CI95), %**	12 (7–20.6)	13.4 (6.7–27.2)	11.1 (3–41)	14.1 (5.9–33.7)	NA	
** 5 years (CI95), %**	10.7 (5.9–19.2)	13.4 (6.7–27.2)	5.6 (8.3–37.3)	9.4 (2.9–30.7)	NA	

ORR: overall response rate; CR: complete remission; CRi: complete remission with incomplete recovery; OS: overall survival; CI: confidence interval.

## Data Availability

The data presented in this study are available in this article and upon reasonable request to the corresponding author.

## Supplementary Materials

The following supporting information can be downloaded at: https://www.mdpi.com/article/10.3390/cancers17152413/s1, Supplementary Table S1: Granular assessment of all cardiac events in the 2L cohort, including type, grade, concomitant azoles, causality, level of care, and outcome. Supplementary Table S2: Multivariate analyses of prognostic factors for development of non-fatal cardiac events in the 2L cohort. Supplementary Table S3: Granular assessment of all cardiac events in the 3L cohort, including type, grade, concomitant azoles, causality, level of care, and outcome.
