# Supplementary material for: Cardiotoxicity in Adult Patients with Relapsed or Refractory Acute Myeloid Leukemia
Source: Cancers (Basel). 2025 Jul 22;17(15):2413. doi: 10.3390/cancers17152413 (PMC12346656; doi:10.3390/cancers17152413)
Supplement: Supplementary file 1 [file cancers-17-02413-s001.zip › cancers-3637473-supplementary.pdf]

Supplementary Materials:

Supplementary Table S1. Granular assessment of all cardiac events in the 2L cohort including: type, grade, concomitant azoles, causality, level of care, and outcome.

Type of cardiac event (CTCAE)	All cardiac events	Grade			Hydroelectrolytic abnormalities		Azole use		Causality			Hospital admission			Outcome	
		1-2	3-4	5	No	Yes	No azole	Azole	Medication	Other	Unknown	Outpatient	Hematology unit	ICU	Resolved	Not resolved
**Total number of events**	207	99	103	5	127	77	86	119	93	79	38	41	141	25	176	31
**Asystole**	1	0	0	1	1	0	0	1	1	0	0	0	1	0	0	1
**Atrial fibrillation**	9	1	8	0	5	3	7	2	2	9	0	0	6	3	7	2
**Cardiac troponin increased**	3	3	0	0	3	0	2	1	1	2	0	0	0	0	2	1
**Cardio-respiratory arrest**	1	0	1	0	1	0	0	1	0	1	0	0	1	0	1	0
**Chest pain - cardiac**	4	3	1	0	3	1	1	3	0	3	1	1	3	0	4	0
**Conduction disorder**	1	1	0	0	1	0	1	0	1	0	0	0	1	0	1	0
**Ejection fraction decreased**	3	2	1	0	1	1	2	0	3	0	0	1	2	0	2	1
**Electrocardiogram QT corrected interval prolonged**	35	31	4	0	18	17	15	20	22	7	6	17	15	3	33	2

**Electrocardiogram T wave abnormal**	2	2	0	0	0	2	1	1	0	2	0	0	1	1	2	0
**Heart failure**	4	0	3	1	1	3	0	4	1	3	0	0	0	4	0	4
**Hypertension**	45	0	45	0	32	13	19	26	16	17	12	8	35	2	37	8
**Hypotension**	1	1	0	0	1	0	1	0	0	1	0	0	1	0	1	0
**Left ventricular systolic dysfunction**	7	0	6	1	6	1	3	3	1	6	0	0	2	5	2	5
**Myocardial infarction**	1	0	1	0	0	1	1	0	0	1	0	0	1	0	1	0
**Pericardial effusion/tamponade**	4	3	1	0	2	1	3	1	0	3	1	1	3	0	2	2
**Presyncope**	12	12	0	0	5	7	0	12	4	4	4	1	11	0	12	0
**Pulmonary edema**	19	1	17	1	8	11	8	11	6	13	0	0	16	3	17	2
**Sinus bradycardia**	39	39	0	0	31	8	13	26	31	2	6	6	33	0	39	0
**Supraventricular tachycardia**	2	0	2	0	2	0	0	2	0	0	2	0	2	0	2	0
**Syncope**	10	0	10	0	6	4	8	2	2	2	6	5	5	0	9	1
**Ventricular fibrillation**	2	0	1	1	0	2	0	2	2	0	0	0	0	2	1	1
**Ventricular tachycardia**	2	0	2	0	0	2	1	1	0	2	0	0	1	1	1	1

Supplementary Table S2. Multivariate analyses of prognostic factors for development of non-fatal cardiac events in the 2L cohort.

		Univariate Analysis	Multivariate Analysis	
**Covariate**	**Unfavorable category**	**P value**	**HR (95% CI)**	**P value**
**Age**	**≥65**	0.36	1 (0.6-1.7)	0.88
**ECOG**	**>2**	0.036	1.4 (0.4-1.4)	0.35
**Cardiologic antecedents (all)**	**Yes**	0.036	1.6 (1.1-2.4)	0.013
**Treatment chemotherapy**	**Intensive**	0.02	1.9 (1.2-3)	0.01
**Clinical trial**	**Yes**	<0.001	2.2 (1.4-3.3)	<0.001
**Use of FLT3 inhibitors**	**No**	0.26	1.8 (0.3-1.1)	0.08

Abbreviations: HR: hazard ratio; CI: confidence interval; FLT3: Fms-like tyrosine kinase 3; ECOG: Eastern oncology cooperative group

Supplementary Table S3. Granular assessment of all cardiac events in the 3L cohort including: type, grade, concomitant azoles, causality, level of care, and outcome.

Type of cardiac event (CTCAE)	All cardiac events	Grade			Hydroelectrolytic abnormalities		Azole use		Causality			Hospital admission			Outcome	
		1-2	3-4	5	No	Yes	No azole	Azole	Medication	Other	Unknown	Outpatient	Hematology unit	ICU	Resolved	Not resolved
Total number of events	159	85	73	1	81	73	76	83	73	73	13	29	118	12	127	32
Atrial fibrillation	14	1	13	0	8	6	7	7	2	11	1	1	11	2	5	9
Atrial flutter	1	0	1	0	0	1	1	0	1	0	0	0	1	0	0	1
Atrioventricular block first degree	1	1	0	0	0	1	0	1	0	1	0	0	1	0	1	0
Cardiac troponin increased	1	1	0	0	0	1	1	0	1	0	0	0	1	0	1	0
Chest pain - cardiac	3	3	0	0	2	1	1	2	0	2	1	0	3	0	3	0
Conduction disorder	2	2	0	0	1	1	2	0	0	1	1	1	1	0	1	1
Ejection fraction decreased	9	7	2	0	4	5	5	4	6	3	0	2	3	4	3	6
Electrocardiogram QT corrected	39	30	9	0	14	23	16	23	23	15	1	9	28	2	34	5

**interval prolonged**																
**Electrocardiogram T wave abnormal**	3	3	0	0	0	3	1	2	0	3	0	0	3	0	3	0
**Hypertension**	30	0	30	0	21	8	15	15	16	11	3	9	21	0	28	2
**Palpitations**	2	2	0	0	2	0	1	1	1	0	1	0	2	0	2	0
**Pericardial effusion/tamponade**	6	5	1	0	4	2	5	1	1	4	1	0	5	1	3	3
**Pericarditis**	2	2	0	0	2	0	0	2	0	2	0	0	2	0	1	1
**Presyncope**	8	8	0	0	4	4	5	3	2	6	0	2	6	0	8	0
**Pulmonary edema**	10	0	9	1	4	5	6	4	4	6	0	0	10	0	8	2
**Right ventricular dysfunction**	1	0	1	0	0	1	0	1	0	0	1	0	1	0	0	1
**Sinus bradycardia**	20	20	0	0	11	9	7	13	16	1	3	5	14	1	20	0
**Supraventricular tachycardia**	1	0	1	0	1	0	1	0	0	1	0	0	0	1	1	0
**Syncope**	6	0	6	0	3	2	2	4	0	6	0	0	6	0	5	1
